# Editorial Comment to A case of tape infection 19 years after insertion of a tension‐free vaginal tape sling

**DOI:** 10.1002/iju5.12478

**Published:** 2022-05-29

**Authors:** Takahito Wakamiya

**Affiliations:** ^1^ Department of Urology Wakayama Medical University Wakayama Japan

Tension‐free vaginal tape (TVT) surgery for stress urinary incontinence is strongly recommended by guidelines, but rarely results in severe complications.^1^ In past reports, TVT has been associated with more bladder perforation than trans‐obturator tape,^2^ which is attributed to the route of tape insertion. Watanabe et al. reported a rare case of tape infection 19 years after insertion of a TVT.^3^ This case has potential importance to alert urologists to keep in mind that TVT surgery can cause severe infection. In the present case, both the tape tips were against the lateral wall of the bladder. It is possible that the tape was already too close to the bladder wall when it was inserted, or that the tape loosened over time and injured the bladder wall. In addition, TVT surgery at a young age could increase the risk of infection in the future because radiation therapy, cancer, and chronic persistent inflammation of the bladder with dysuria can increase the risk of tape infection and abscess formation.

Obviously, the crucial point is to determine the indications for TVT surgery, including age, medical complications, and of surgical history, and to insert the tape in the appropriate position by the appropriate route. As for follow‐up, we believe that abdominal ultrasonography, including residual urine measurement, should be continued in consideration of long‐term complications after TVT surgery.

## Conflict of interest

The authors declare no conflicts of interest.
